# Low Dose Medicine: theoretical background and scientific evidence

**DOI:** 10.1186/s13052-018-0460-6

**Published:** 2018-02-08

**Authors:** S. Bernasconi

**Affiliations:** Former Director Pediatric Department, University of Parma, Via A. Catalani 10, 43123 Parma, Italy

## Introduction

The paper recently published by Carello R. et al. *Long-term treatment with Low Dose Medicine in chronic childhood eczema. A double-blind two-stage randomized clinical trial* (IJP 2017 43:78) highlights a new possible therapeutic approach for the treatment of chronic childhood eczema. It is based on the oral administration of low doses activated interleukin 12 and Interferon-γ in order to restore the immunologic balance, altered in the disease. This short commentary will briefly describe the theoretical background of Low Dose Medicine (LDM), with particular focus on the aspects linked with the efficacy and the safety of the LDM treatment.

### The origin of LDM

LDM was born from the convergence between molecular biology studies (MB) and psycho-neuro-endocrine-immunology (P.N.E.I.) and was initially developed from the research results in the field of nanopharmacology.

Thanks to MB it is clear today of the role played by a significant number of molecules defined by the collective name of signaling (or messenger) molecules, which can drive both intra- and extracellular signaling pathways.

These molecules include neuropeptides, hormones, and cytokines, along with growth factors, and are fundamental regulatory molecules for cellular and tissue functions.

The main unifying P.N.E.I. element is identified in the recognized centrality of the cross-talk between the psychoneuroendocrine systems and the immune system [1–4].

This cross-talk is mediated by a network of signaling molecules which provides the correct biological information for the regulation of cellular responses to internal and external stimuli. An impaired (or altered) cross-talk, due to an imbalance between specific signaling molecules, is fundamental, for example, in inflammatory, allergic and autoimmune diseases onset [5–7]; preserving and restoring the physiological levels of messenger molecules is the goal for a possible new therapeutic strategy aimed to the recovery of the homeostatic equilibrium.

In homeostatic (healthy) conditions, in fact, the levels of these molecules in the extra-cellular matrix [8, 9] physiologically fluctuate in a specific range (from nanograms/ml to femtograms/ml); on the contrary, every particular pathologic condition can be considered as the expression and consequence of changed concentrations of specific signaling molecules [10–13].

The use of biological molecules, which control and drive cellular functions in order to restore the initial physiological conditions, is the core of LDM.

It is necessary to focus attention on several aspects related to the P.N.E.I. network management, which are cardinal for a deep understanding of Low Dose Medicine:

- P.N.E.I. cross talk is bi-directional, as are the effects of the alteration of the cross talk itself [14–16].
- Intercellular signaling is regulated by the diffusion of messenger molecules in the extracellular matrix (ECM); pathological alteration of the ECM reverberates on an impairment of the communication between cells, organs and systems [17, 18].
- Ligands-receptors interaction is a key modulating factor of the signal transduction: substrate concentration and binding affinity and saturation properties are key parameters [19, 20].

The opportunity to balance specific alterations of the immune system with the use of cytokines, or to balance endocrine disorders with the use of hormones, represents one of the most intriguing research fields in Biomedical Science, but unfortunately the clinical application of this concept collides with the severe dose-dependent adverse effects that these substances demonstrate when utilized at the usual and common pharmacological concentration (not low dose, which is sub-nanomolar).

The physiological low doses of signaling molecules, which represent the LDM therapeutic tools, are orally administered and their activity is systemic. Scientific literature recognizes that the oral intake of cytokines is effective in modulating the immune response [21–23].

A possible action mechanism for orally administered peptides involves M cells at the intestinal epithelium level. Signaling molecules are detected by M cells directly in the intestinal lumen and presented to immune T cells within Peyer’s patches lymph nodes [24] inducing a specific immune response.

A possible pitfall of signaling molecules (and peptides in general) oral administration, when used in low dose, is exhibited by their low bioavailability (typically less than 1–2%): an effective drug delivery system is required to overcome this problem.

The effective oral administration of signaling molecules at physiological low doses (nanograms-femtograms) is made possible by the application of SKA technology (Sequential Kinetic Activation), an innovative drug delivery system, based on the principle of release activity (the capability of the basic substance to release its pharmacological activity in the water milieu) [25] that allows the sub-nanomolar concentrations to be active even below the actually considered minimum effective dose with therapeutic results comparable to those induced by high concentrations.

The action mechanism of low dose SKA cytokines, hormones, neuropeptides and growth factors involves the sensitization (or activation) of cellular and plasmatic receptors by virtue of their high dilution, [physiological working range between 10^− 6^ (microgram) for hormones [8] and 10^− 15^ (femtogram) for the other messenger molecules] [9].

On the basis of these premises, the possible clinical therapeutic LDM approaches are:

1. To restore the P.N.E.I. homeostasis by stimulating a pathologically impaired (down-regulated) cellular pathway using the same cytokines, hormones, neuropeptides or growth factors (low dose SKA) which are physiologically involved in the cellular signaling;
2. To use antagonistic molecules (low dose SKA) in order to rebalance the levels of pathologically up-regulated molecules via negative feedback mechanisms.

### Low Dose Medicine and scientific research

Approximately ten years of scientific research in the field of LDM have shown the validity of the conceptual approach and the effectiveness and safety of the therapeutic intervention based on the oral administration of sub-nanomolar low doses of signaling molecules (Table 1) [26–41].

**Table 1:** Most important peer reviewed published papers in the last 10 years

Year	Authors	Journal	Study Type	Title	Molecules and Tested Drugs
2009	Gariboldi S. et al. [26]	Pulmonary Pharmacology & Therapeutics	In vivo basic research	Low dose oral administration of cytokines for treatment of allergic asthma	IL-12 IFN-γ
2012	D’amico L. et al. [27]	Journal of Cancer Therapy	Ex vivo basic research	Low Dose of IL-12 stimulates T Cell response in cultures of PBMCs derived from Non-Small Cell Lung Cancer Patients	IL-12
2013	Cardani D. et al. [28]	Gastroenterology Research	In vivo basic research	Oral Administration of Interleukin-10 and Anti-IL-1 Antibody Ameliorates Experimental Intestinal Inflammation.	IL-10 Antibodies-anti IL-1
2014	Radice E. et al. [29]	International Immunopharmacology	Ex vivo basic research	Low-doses of sequential-kinetic-activated interferon-gamma enhance the ex vivo cytotoxicity of peripheral blood natural killer cells from patients with early-stage colorectal cancer. A preliminary study	IFN-γ
2014	Roberti ML. et al. [30]	Journal of Biological Regulatory & Homeostatic Agents	Clinical trial	Immunomodulating treatment with low dose Interleukin-4, Interleukin-10 and Interleukin-11 in psoriasis vulgaris.	IL-4 IL-10 IL-11
2015	Luchetti P. [31]	Minerva Medica Oftalmologica	Clinical trial	Increasing of visual function in patients with retinal atrophy treated with drugs of Low Dose Medicine. Monocentric retrospective observational study.	NT3 NT4 NGF Retina suis Injeel Solanum compositum Ubichinon compositum
2015	Barygina V. et al. [32]	Journal of Dermatological Science	In vitro basic research	Treatment with low-dose cytokines reduces oxidative-mediated injury in perilesional keratinocytes from vitiligo skin.	IL-4 IL-10 b-FGF β-endorphin
2015	Lotti T. et al. [33]	Journal of Biological Regulatory & Homeostatic Agents	Clinical trial	Vitiligo: successful combination treatment based on oral low dose cytokines and different topical treatments.	IL-4 IL-10 Antibodies-anti IL-1 b-FGF
2015	Radice E. et al. [34]	Translational Oncology	Ex vivo basic research	Enhancement of the Immunostimulatory Functions of Ex Vivo-Generated Dendritic Cells from Early-Stage Colon Cancer Patients by Consecutive Exposure to Low Doses of Sequential-Kinetic-Activated IL-4 and IL-12.	IL-4 IL-12
2015	Lotti T. et al. [35]	Der Hautarzt	Clinical trial	Successful combination treatment for psoriasis with phototherapy and low-dose cytokines: A spontaneous, retrospective observational clinical study.	IL-4 IL-10 Antibodies-anti IL-1
2016	Barygina V. et al. [36]	Journal of Dermatological Science	In vitro basic research	Low dose cytokines reduce oxidative stress in primary lesional fibroblasts obtained from psoriatic patients.	IL-4 IL-10 b-FGF β-endorphin
2016	Fiorito F. et al. [37]	Comparative Immunology, Microbiology and Infectious Diseases	Clinical trial (veterinary)	Clinical improvement in feline herpesvirus 1 infected cats by oral low dose of interleukin-12 plus interferon-gamma.	IL-12 IFN-γ
2016	Genazzani A. et al. [38]	Bollettino di Ginecologia Endocrinologica Frontiers in Gynecological Endocrinology	Observational pilot study	Pharmacological and Integrative Treatment of Stress-Induced Hypothalamic Amenorrhea	Beta-Estradiol
2017	Martin Martin LS. et al. [39]	Drug Design, Development and Therapy	Clinical trial	An open randomized active-controlled clinical trial with low-dose SKA cytokines versus DMARDs evaluating low disease activity maintenance in patients with rheumatoid arthritis.	Antibodies-anti IL-1 IL-10 IL-4
2017	Castiglioni S. et al. [40]	International Journal of Molecular Sciences	In vitro basic research	Femtograms of Interferon- γ Suffice to Modulate the Behavior of Jurkat Cells: A New Light in Immunomodulation	IFN-γ
2018	Mancini F. et al. [41]	International Immunopharmacology	In vitro basic research	Low-dose SKA Progesterone and Interleukin-10 modulate the inflammatory pathway in endometriotic cell lines	Progesterone IL-10

More precisely, since 2009, 8 basic in vitro*/*ex vivo research, 4 basic in vivo (murine model) research, and 10 clinical trials have been published.

In the field of basic in vitro*/*ex vivo research, the study *Low-dose SKA Progesterone and Interleukin-10 modulate the inflammatory pathway in endometriotic cell lines* (Mancini F. et al. *Int Immunopharmacol.* 2018;55:223–230) is paradigmatic because it shows the intimate action mechanisms of low dose cytokines and hormones in the modulation of inflammatory phenomena.

The aim of the study performed by Mancini F. and colleagues was to evaluate the efficacy of low-dose SKA Progesterone and IL-10 in the modulation of the inflammatory response in endometriotic cell lines. The results demonstrate that progesterone low-dose SKA is effective in the modulation of HSD17B1 protein levels and of NF-kB activity; IL-10 low-dose activated is effective in the inhibition of NF-kB activity; the combination of low-dose activated Progesterone and IL-10 shows an additive effect on the inhibition of NF-kB activity.

In the field of basic in vivo (murine model) research, the study *Low dose oral administration of cytokines for treatment of allergic asthma* (Gariboldi S. et al. *Pulm Pharmacol Ther* 2009;22(6):497–510) is paradigmatic because it shows the ability of low doses activated proteins to induce a cellular response and, consequently, to enhance the endogenous expression of the same proteins.

The study is based on the fact that allergic patients typically show a hyper-expression of Th2 lymphocytes and, therefore, an over synthesis of two cytokines produced by these lypmphocytes: Interleukin-4 (IL-4) and Interleukin-5 (IL-5). The study results show that the in vivo administration of low-dose SKA IL-12 and IFN-γ is successful, producing an increase in IL-12 and IFN-γ blood levels (which are able to down-regulate Th2 lymphocytes) and, at the same time, a decrease in IL-4 and IL-5 blood levels.

In the field of clinical research, the study *An open randomized active-controlled clinical trial with low-dose SKA cytokines* versus *DMARDs evaluating low disease activity maintenance in patients with rheumatoid arthritis.* (Matrin Martin LS. et al. *Drug Des Devel Ther.* 2017;11:985–994) is paradigmatic because it represents a perfect example of *overlapping* therapy between low dose pharmacology and conventional “gold standard” treatments.

The study suggests that the therapy with low-dose SKA cytokines/antibodies in patients with Rheumatoid Arthritis in remission is able to keep low the disease activity by inducing a progressive regulation and stabilization of the Immune System. The published results provide valuable data regarding the immunomodulatory activity of a combination therapy with low- dose SKA IL-4 plus IL-10 plus Anti IL-1 antibodies and indicate novel therapeutic approaches for Rheumatoid Arthritis control (as well as for other autoimmune diseases) even in the era of biologics.

Today, we can state that scientific literature supports the therapeutic approach of LDM and that it is no longer only a scientific theory, but it can be the basis for a new medical paradigm, particularly in the pediatric field.

In the last ten years, the available scientific literature suggests to persevere in this direction, especially given the high safety and effectiveness of low dose drugs in maintaining a low disease activity rate in patients with complex pathological pictures. Low dose therapies are particularly useful in long-term treatments, due to the absence of adverse effects and overloading phenomena.

However some limitations have been highlighted, such as the fact that in highly compromised situations, where homeostatic and biological regulation systems are deeply modified, low dose pharmacology cannot reach the hoped-for effects.

In the near future, Low Dose Medicine will help us understand how to act on the most intimate causes of many pathologies - especially inflammatory - that recognize the origin of altered immune system communication. It cannot be incidental that severe and increasing incidence of diseases, such as intestinal inflammatory diseases, rheumatoid arthritis, psoriasis, atopic dermatitis, allergies, and even osteoarthritis, are all commonly associated with inflammation.

## Conclusion

Less than ten years have passed since the first publication on low-dose cytokine activity at sub-nanomolar dosages appeared.

A decade of research on Low Dose Medicine and low dose Pharmacology empowered the researchers to collect a scientifically relevant critical mass of basic and clinical data able to demonstrate:

1. The validity of the concepts underpinning the LDM theoretical approach.
2. The necessary role of the pharmaceutical technological process called SKA.
3. The preclinical and clinical effectiveness of low dose SKA activated signaling molecules.
4. The effectiveness of low dose drugs in maintaining a low disease activity rate.
5. The safety of the tested molecules, which enables chronic or long-term treatments.

Now, that the interest of the scientific community on low dose pharmacology is very high [42, 43], it is necessary to proceed with further insights and the acquisition of evidence through solid research for the definitive affirmation of LDM in daily clinical practice.
